# Effectiveness and safety of finerenone in Chinese CKD patients without diabetes: a retrospective, real-world study

**DOI:** 10.1007/s11255-024-04142-1

**Published:** 2024-07-10

**Authors:** Li Zhou, Wenge Li

**Affiliations:** Department of Nephropathy, China-Japan Friendship Hospital, Beijing, 100029 China

**Keywords:** Finerenone, Chronic kidney disease, Non-diabetic, Effectiveness

## Abstract

**Background:**

Finerenone, a non-steroidal mineralocorticoid receptor antagonist, has previously demonstrated its efficacy and safety in chronic kidney disease (CKD) associated with diabetes mellitus. Given its therapeutic potential, finerenone has been preliminarily explored in clinical practice for non-diabetic CKD patients. The effectiveness and safety in this population require further investigation in a real-world setting.

**Methods:**

This retrospective, real-world analysis included non-diabetic CKD patients receiving finerenone. The main clinical outcomes assessed were changes in urinary albumin-to-creatinine ratio (UACR) and estimated glomerular filtration rate (eGFR). Serum potassium (sK^+^) levels were also monitored. Data were collected at baseline, and then at 1 month and 3 months following treatment initiation.

**Results:**

Totally, 16 patients were included. There was a notable decrease in UACR from 1-month post-treatment, with a further reduction at 3 months, resulting in a median reduction of 200.41 mg/g (IQR, 84.04–1057.10 mg/g; *P* = 0.028; percent change, 44.52% [IQR, 31.79–65.42%]). The average eGFR at baseline was 80.16 ml/min/1.73m^2^, with no significant change after 1 month (80.72 ml/min/1.73m^2^, *P* = 0.594) and a slight numerical increase to 83.45 ml/min/1.73m^2^ (*P* = 0.484) after 3 months. During the 3-month follow-up, sK^+^ levels showed only minor fluctuations, with no significant differences compared to baseline, and remained within the normal range throughout the treatment period. No treatment discontinuation or hospitalization due to hyperkalemia was observed.

**Conclusion:**

In non-diabetic CKD patients, finerenone showed good effectiveness and safety within a 3-month follow-up period. This study provides valuable real-world evidence supporting the use of finerenone in non-diabetic CKD and highlights the need for future large-scale prospective research to further validate its efficacy.

**Supplementary Information:**

The online version contains supplementary material available at 10.1007/s11255-024-04142-1.

## Introduction

Chronic kidney disease (CKD) represents a global public health concern, characterized by a gradual loss of renal function over time [1]. CKD affects approximately 10% of the worldwide population, with a higher prevalence observed in aging societies [2]. This condition is inherently linked to several unfavorable consequences, including an elevated susceptibility to cardiovascular disease, heightened rates of hospitalization, and increased mortality, thereby imposing a significant strain on healthcare systems worldwide [3]. In the pathophysiology of CKD, the overactivation of mineralocorticoid receptors (MR) has been implicated as a pivotal factor contributing to renal inflammation and fibrosis [4]. The harmful repercussions of excessive MR activation extend beyond sodium retention and the onset of hypertension, triggering a series of inflammatory and fibrotic responses in the renal tissue, which further exacerbate the decline in renal function [5].

The utilization of mineralocorticoid receptor antagonists (MRAs) has emerged as a promising therapeutic strategy, offering renal protection through multifaceted mechanisms [6]. These agents attenuate the inflammatory milieu by inhibiting the release of pro-inflammatory mediators and cytokines. They also impede collagen deposition in the kidney compartments and alleviate macrophage infiltration, collectively attenuating the progression of renal fibrosis [4]. Furthermore, MRAs have been shown to inhibit the epithelial-mesenchymal transition (EMT), a critical process in renal fibrosis [7, 8]. MRAs are categorized into steroid-based compounds, such as spironolactone and eplerenone, and non-steroidal agents, including finerenone and esaxerenone [9]. Steroid-based MRAs have been pivotal in treating heart failure and have shown promise in retarding the progression of renal dysfunction. However, their use in CKD management is limited by the potential risk of hyperkalemia and the challenge of balancing MR activity and selectivity [10]. Non-steroidal MRAs have gained attention for their kidney-protective potential, offering a compelling addition to CKD treatment [11]. Their ability to modulate MR activity without the limiting side effects of steroid-based MRAs represents a significant advancement in CKD management [12].

Finerenone, a non-steroidal MRA, is characterized by a high affinity and selectivity for MR. Structurally refined from a naphthalene base and developed based on the dihydropyridine architecture, finerenone effectively impedes the recruitment of transcription co-activators. This targeted mechanism of action specifically ameliorates the inflammatory cascade and fibrotic processes within the kidneys [13, 14]. At present, finerenone is predominantly utilized in the management of CKD associated with type 2 diabetes mellitus (T2DM). The ARTS-DN trial demonstrated notable treatment efficacy of finerenone, as indicated by the significant dose-dependent reduction in the urinary albumin–creatinine ratio (UACR) in diabetic nephropathy patients, reinforcing its renal-protective capabilities [15]. The FIDELIO-DKD trial, which included 5,734 patients with CKD and T2DM, most of whom were in CKD stage 3 and above (88.4%), revealed that treatment with finerenone, compared to placebo, significantly reduced the risks of CKD progression and cardiovascular events in this population [16]. Similar findings were shown in the China subgroup study [17]. In accordance with these findings, the FIGARO-DKD trial also indicated that finerenone resulted in a lower rate of a composite outcome, including kidney failure, sustained decrease from baseline of at least 40% in the estimated glomerular filtration rate (eGFR), or death from renal causes, compared with placebo in patients with CKD and T2DM [18]. Notably, more than half of the patients (61.7%) in the FIGARO-DKD trial were in CKD stages 1 and 2. These findings suggest that finerenone has beneficial cardiovascular and renal-protective effects, along with a good safety profile, across all stages of T2DM CKD patients. This is further supported by the FIDELITY pooled study [19]. Furthermore, a meta-analysis incorporating data from four trials has demonstrated substantial advantages in reducing renal composite outcomes, decreasing UACR, and preventing eGFR decline in T2DM patients with established CKD following finerenone treatment [20]. The promising outcomes of finerenone trials suggest its transformative potential in reshaping the landscape of therapeutic interventions for CKD.

Finerenone has demonstrated promising therapeutic effects and safety in diabetic CKD, yet the scope of CKD extends beyond diabetes-related conditions, including those caused by hypertension and glomerulonephritis [21]. Non-diabetic CKD differs from diabetic CKD in its underlying pathogenesis. While diabetic CKD is primarily driven by hyperglycemia-induced damage leading to glomerulosclerosis and tubulointerstitial fibrosis, non-diabetic CKD can arise from various etiologies, such as hypertension, glomerulonephritis, and polycystic kidney disease [21]. These differences underscore the need to explore the efficacy and safety of treatments specifically in non-diabetic CKD populations. Other MRAs, such as spironolactone and eplerenone, have demonstrated cardio-renal-protective effects in non-diabetic CKD. For instance, studies have shown that eplerenone reduces proteinuria and blood pressure in non-diabetic CKD patients [22]. These findings highlight the potential benefits of MRAs in this patient population and emphasize the need for more data on finerenone's efficacy in non-diabetic CKD. Given the current lack of efficacy data on finerenone in non-diabetic CKD, it is crucial to investigate its effects in this specific patient group.

A notable randomized-controlled trial exploring finerenone in patients with chronic heart failure and CKD (including non-diabetic CKD) showed that finerenone also led to significantly reduced UACR levels and lower rates of renal failure, providing insights into its broader applicability [23]. Despite these advances, clinical evidence elucidating finerenone's use in non-diabetic CKD remains insufficient. There is an ongoing phase 3 randomized, double-blind, placebo-controlled trial, namely the FIND-CKD trial (NCT05047263), poised to expand our understanding of finerenone's role in preventing kidney disease progression in a non-diabetic setting. Nevertheless, the results of this study are yet to be announced. In line with these considerations, we performed a retrospective, real-world study aiming to investigate finerenone's effectiveness and safety in Chinese non-diabetic CKD patients.

## Methods

### Study design and patients

This retrospective, real-world study was conducted in the Department of Nephrology, China–Japan Friendship Hospital. Patients from November 2022 to August 2023 were screened. Inclusion criteria were (1) patients aged 18–85 years; (2) patients diagnosed with CKD stages 1–4 according to the 2012 Kidney Disease: Improving Global Outcomes (KDIGO) guideline [24]; (3) patients receiving finerenone for CKD treatment, (4) patients with a minimum follow-up duration of no less than 3 months; (5) patients using or not using renin-angiotensin system inhibitors (RASi) were both eligible for inclusion. Exclusion criteria were (1) patients with diagnosis of diabetes mellitus, or Addison's disease; (2) patients with significantly incomplete medical records, which could compromise the integrity of the assessment; (3) patients presenting with insufficient liver function of Child–Pugh C.

This study was reviewed and approved by the Ethics Committee of the China–Japan Friendship Hospital. Given its retrospective nature, the requirement for written informed consent in this study was waived by the Ethics Committee.

## Treatment strategy

Patients received standard care for CKD based on the disease type and associated comorbidities, such as hypertension, abnormal lipid metabolism, arteriosclerosis obliterans, and hyperuricemia. Treatment decisions were tailored to each patient's specific clinical profile.

For finerenone therapy, the initial dosing was determined based on the patient’s eGFR. For patients with an eGFR ≥ 60 mL/min/1.73m^2^, the starting dose of finerenone was set at 20 mg per day. For those with an eGFR ranging from 25 to 60 mL/min/1.73m^2^, a reduced initial dose of 10 mg per day was administered. Finerenone was not used for patients with an eGFR < 25 mL/min/1.73m^2^. Dose adjustments were made according to the monitoring of sK^+^ levels and eGFR values throughout the treatment period. Dose escalation to 20 mg per day was considered when sK^+^ levels were ≤ 4.8 mmol/L. The dose was maintained if sK^+^ levels ranged between 4.8 and 5.5 mmol/L. If sK^+^ levels exceeded 5.5 mmol/L, the use of finerenone was suspended. Treatment could be resumed, starting at a reduced dose of 10 mg per day, once sK^+^ levels decreased to ≤ 5.0 mmol/L.

## Data collection and outcomes

In this retrospective study, baseline demographic data and disease characteristics of CKD patients were collected. The main therapeutic outcomes were UACR and eGFR. The key safety outcome assessed was sK^+^ level. In clinical practice, patients receiving finerenone undergo comprehensive laboratory tests when starting treatment and are routinely re-examined at 1 month and 3 months post-treatment as per medical advice. Therefore, data were retrospectively collected at three time points: baseline (at the initiation of finerenone treatment), 1 month after treatment initiation, and 3 months after treatment initiation. UACR was measured by the Mindray BS800M automatic biochemical analyzer (Mindray Bio-medical Electronics Co., Ltd., Shenzhen, China), using the immunoturbidimetric method with reagents provided by Beijing Leadman Biochemistry Co., Ltd. eGFR was calculated using the CKD-EPI formula, which is based on serum creatinine levels, gender, and age [25].

## Statistical analysis

In this study, categorical data were presented as frequencies and percentages. The normality of continuous data was assessed using the Shapiro–Wilk test. Normally distributed data were described using mean ± standard deviation (SD), while skewed data were presented as median and interquartile range (IQR). Repeated measures were analyzed using ANOVA or generalized estimating equations, depending on the results of the normality test. Subgroup analyses were conducted based on the use of RASi. Statistical significance was set at a two-tailed α of 0.05. All analyses were performed using R version 4.3.0.

## Patient and public involvement

Nor patients or the public were involved.

## Results

### Patient characteristics

The study included 16 patients, predominantly male (75.0%), with a mean age of 55.38 ± 14.37 years. As shown in Table 1, the types of kidney disease included clinical diagnoses such as chronic nephritis in 50% of patients and nephrotic syndrome in 12.5%. Etiological diagnoses included IgA nephropathy and membranous nephropathy (each accounting for 12.5%), polycystic kidney disease (6.3%), and Henoch–Schönlein purpura nephritis (6.3%).

**Table 1:** Demographic and baseline characteristics of the patients

Characteristics	All (*N* = 16)
Age (years)	55.38 ± 14.37
Male sex	12 (75.0%)
Kidney diseases (clinical diagnosis)	
Chronic nephritis	8 (50%)
Nephrotic syndrome	2 (12.5%)
Kidney diseases (etiological diagnosis)	
IgA nephropathy	2 (12.5%)
Membranous nephropathy	2 (12.5%)
Polycystic kidney disease	1 (6.3%)
Henoch–Schönlein purpura nephritis	1 (6.3%)
Baseline UACR (mg/g)	643.58 (187.52, 2254.41)
≤ 300	4 (25%)
> 300	7 (43.8%)
Baseline eGFR (ml/min/1.73m^2^)	80.16 ± 31.46
25–45	3 (18.8%)
45–60	1 (6.3%)
> 60	10 (62.5%)
Baseline serum potassium (mmol/L)	4.38 ± 0.72
Concomitant medications	
RASi	8 (50%)
Diuretics	4 (25%)
Steroid	1 (6.3%)
Chinese traditional medicines	11 (68.8%)
Antithrombotic/anticoagulant drugs	12 (75%)
Statins	6 (37.5%)
Allopurinol/benzbromarone	3 (18.8%)
Antianemic drugs	4 (25%)

*UACR* urinary albumin-to-creatinine ratio, *eGFR* estimated glomerular filtration rate; *RASi* renin-angiotensin system inhibitors

Baseline UACR had a median value of 643.58 mg/g (IQR, 187.52–2254.41). Regarding renal function, the average eGFR at baseline was 80.16 ± 31.46 mL/min/1.73m^2^. The mean baseline serum potassium level was 4.38 ± 0.72 mmol/L (Table 1).

## Treatment pattern

Twelve patients initiated therapy with a daily dose of 10 mg, while 4 patients started at a higher dose of 20 mg per day. During the treatment period, 6 patients who initially received 10 mg per day had their dose adjusted to 20 mg. Ultimately, a total of 10 patients were administered finerenone at the target dose of 20 mg daily. For concomitant medications, the most commonly used were antithrombotic/anticoagulant drugs (75%), traditional Chinese medicines (68.8%), RASi (50%), and statins (37.5%). Other medications included diuretics, anti-anemia agents, allopurinol, benzbromarone, and steroids (Table 1).

## Therapeutic outcomes

**Fig. 1:**
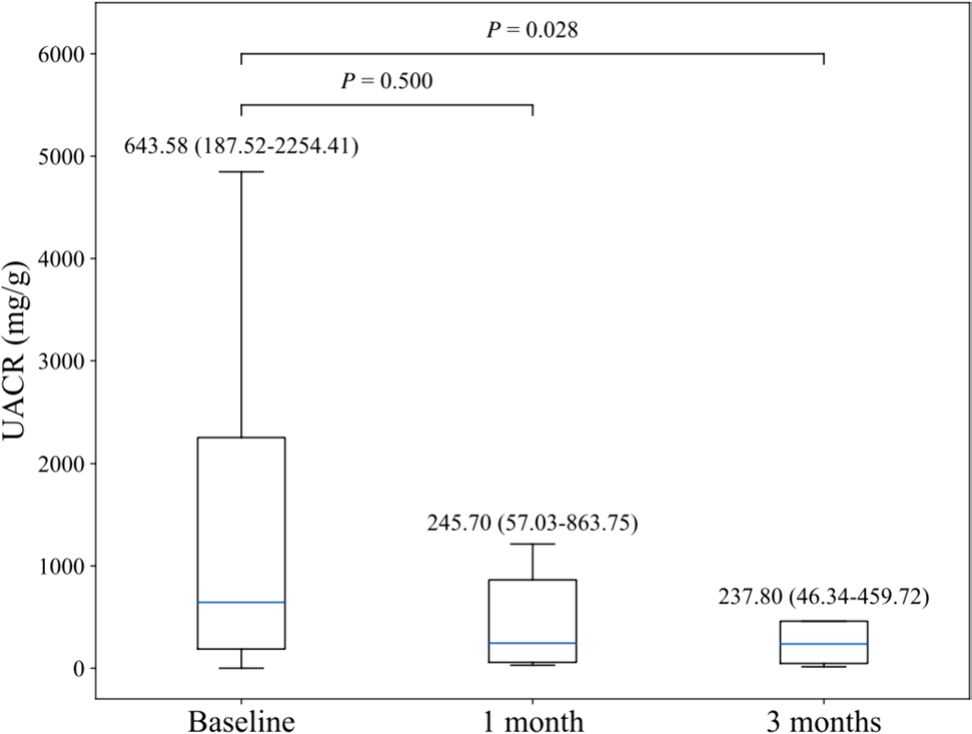
Changes in urinary albumin-to-creatinine ratio (UACR) over time. Data are presented as median and interquartile range

As shown in Table 2 and Fig. 1, UACR showed a remarkable decrease after 1 month of treatment, and a significant reduction of 200.41 mg/g (IQR, 84.04–1057.10; P = 0.028) was further observed at the 3-month follow-up. The reduction percentage of UACR was 44.52% (IQR, 31.79%-65.42%) at 3 months.

**Table 2:** Clinical measurements at baseline, 1 month, and 3 months

Variables	Baseline	Month 1	Difference from baseline	*P*	Month 3	Difference from baseline	*P*
UACR (mg/g)				0.500			0.028
Mean ± SD	1691.90 ± 2322.52	985.80 ± 1726.91	52.26 ± 300.20		632.46 ± 1132.56	− 536.16 ± 647.29	
Median (IQR)	643.58 (187.52, 2254.41)	245.70 (57.03, 863.75)	− 21.37 (− 62.91, − 14.85)		237.80 (46.34, 459.72)	− 200.41 (− 1057.10, − 84.04)	
Range	(0.11, 7022.48)	(28.96, 4784.42)	(− 210.01, 570.44)		(14.41, 3510.18)	(− 1395.43, -32.78)	
eGFR (ml/min/1.73m^2^)				0.594			0.484
Mean ± SD	80.16 ± 31.46	80.72 ± 36.41	0.72 ± 5.01		83.45 ± 26.89	− 0.40 ± 10.55	
Median (IQR)	90.25 (54.57, 100.37)	97.64 (38.63, 100.02)	0.33 ( − 2.73, 3.71)		90.86 (75.36, 101.79)	− 1.54 (− 5.08, 1.46)	
Range	(26.37, 122.98)	(29.13, 120.25)	( − 6.39, 10.22)		(27.07,109.65)	(− 15.17, 21.64)	
Serum potassium (mmol/L)				0.799			0.944
Mean ± SD	4.38 ± 0.72	4.51 ± 0.46	− 0.04 ± 0.91		4.38 ± 0.42	− 0.23 ± 1.00	
Median (IQR)	4.30 (3.93, 4.48)	4.40 (4.20, 4.80)	0.00 (− 0.60, 0.60)		4.50 (4.00,4.70)	0.05 (− 0.40, 0.32)	
Range	(3.60, 6.10)	(3.90, 5.30)	(− 1.40, 1.20)		(3.80, 4.90)	(− 2.30, 0.90)	

Data at 1 month and 3 months were analyzed compared with baseline using the Wilcoxon signed-rank sum test due to non-normal distribution. Differences from baseline represent changes without imputation for missing values. *UACR* urinary albumin-to-creatinine ratio, *eGFR* estimated glomerular filtration rate, *SD* standard deviation, *IQR* interquartile range

**Fig. 2:**
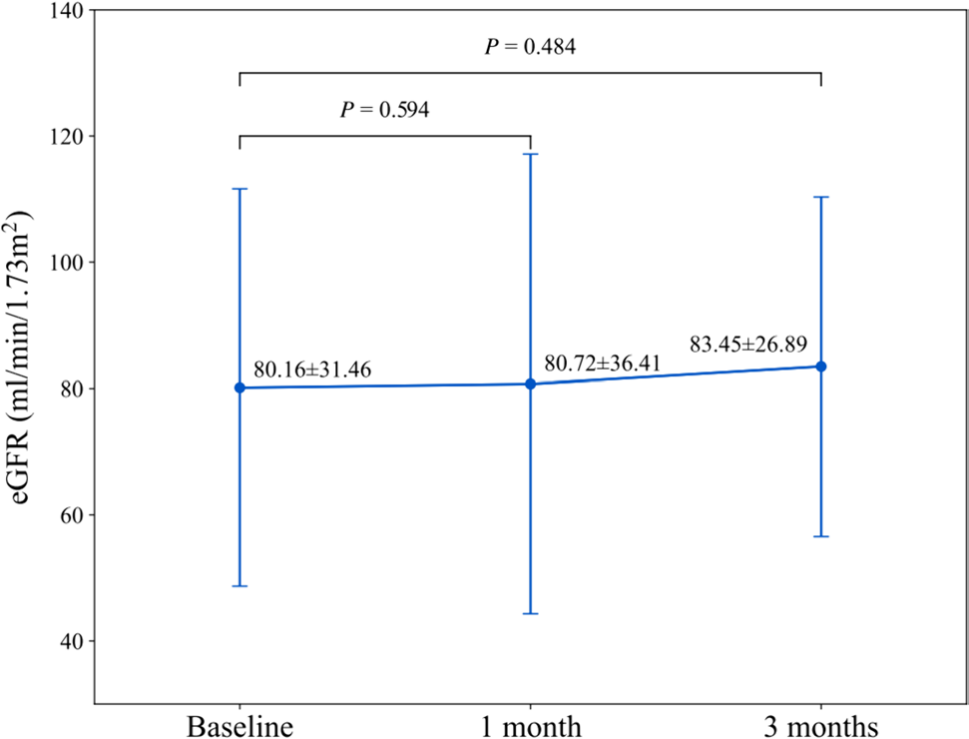
Changes in estimated glomerular filtration rate (eGFR) over time. Data are presented as mean ± standard deviation

Regarding eGFR, no obvious alteration was observed after 1 month of treatment (80.72 ± 36.41 ml/min/1.73m^2^, *P* = 0.594), as shown in Table 2 and Fig. 2. At the 3-month follow-up, there was a minor numerical increase in the mean eGFR value without statistical significance (83.45 ± 26.89 ml/min/1.73m^2^, *P* = 0.484).

In both the concomitant RASi subgroup and the non-RASi subgroup (Supplemental Table 1, Supplemental Fig. 1), UACR demonstrated a remarkably progressive decrease within the 3-month follow-up, indicated by the reduction at the 1-month follow-up and a pronounced decline observed after 3 months. After 3 months of treatment, the reduction percentage of UACR from baseline was 34.51% (IQR, 31.05%-37.97%) in patients without RASi treatment and 59.48% (IQR, 42.85%-77.4%) in those receiving RASi. On the other hand, eGFR showed only minor fluctuations with no significant difference from baseline in both the concomitant RASi subgroup and the non-RASi subgroup (Supplemental Table 2, Supplemental Fig. 2).

## Safety

**Fig. 3:**
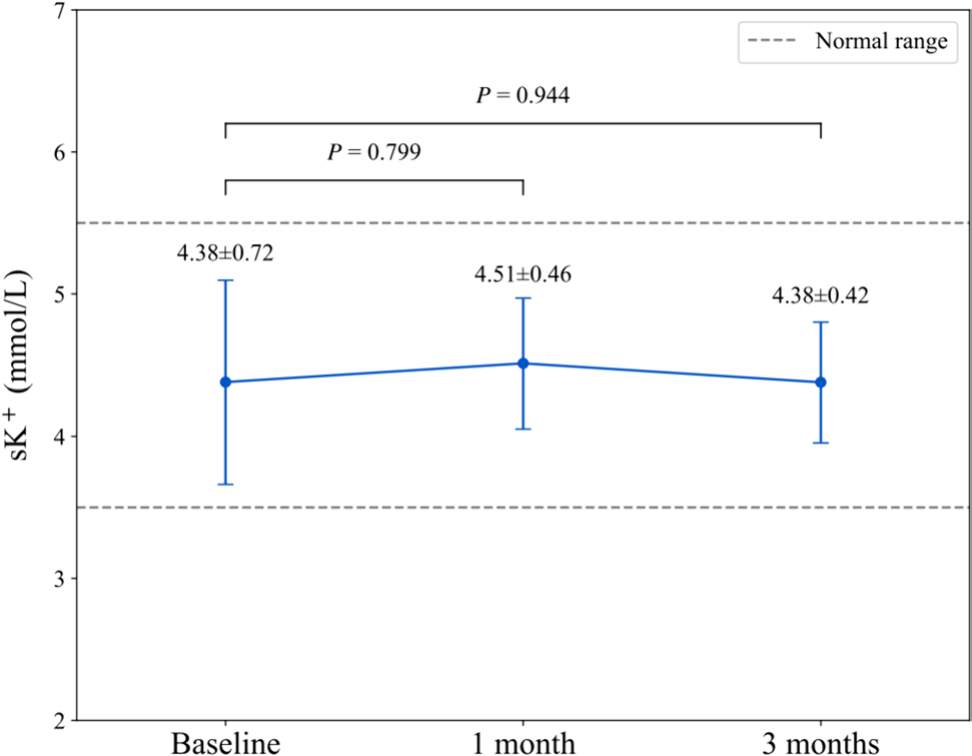
Changes in serum potassium over time. Data are presented as mean ± standard deviation

Throughout the study, sK^+^ levels were maintained within the normal range of 3.5–5.5 mmol/L, with only minor fluctuations observed (Table 2 and Fig. 3). At the 1-month follow-up, sK^+^ levels showed a change of – 0.04 ± 0.91 mmol/L from baseline. After 3 months, the mean sK^+^ level was 4.38 ± 0.42 mmol/L, which was similar to the baseline level (*P* = 0.944). No treatment discontinuation or hospitalization due to hyperkalemia was reported.

## Discussion

This study serves as the first real-world report examining finerenone’s use in non-diabetic CKD patients, marking a significant advancement in understanding the drug's impact. Our findings revealed finerenone's promising potential, evidenced by a noteworthy reduction in UACR and stable eGFR throughout the treatment period, suggesting its capacity to mitigate renal deterioration. Another pivotal aspect of this study is the safety profile of finerenone in the non-diabetic CKD cohort. sK^+^ levels remained relatively stable throughout the treatment, with no instances of treatment discontinuation or hospitalization due to hyperkalemia, underscoring the low risk of hyperkalemia associated with finerenone. Despite emerging clinical applications of finerenone in this patient population, previous clinical evidence has been markedly limited. Our study provides preliminary but valuable insights into the effectiveness and safety of finerenone in non-diabetic CKD, contributing critical clinical evidence to support its use.

The exploration of finerenone's role in the management of non-diabetic CKD is of paramount importance. However, despite encouraging results as indicated by previous study [23], existing evidence remains inconclusive, warranting a more nuanced understanding of its specific therapeutic benefits and safety profile in this particular patient population. A decrease in UACR in CKD patients signifies an improvement in renal function and a reduction in renal disease progression. Studies have shown that reductions in UACR are associated with better clinical outcomes [26]. The significant decrease in UACR observed in our study suggests that finerenone effectively ameliorates renal function in non-diabetic CKD patients. Preclinical studies have demonstrated that finerenone exerts its renoprotective effects through anti-inflammatory and anti-fibrotic actions [27, 28], contributing to the observed UACR reduction. Previous studies have shown similar reductions in UACR with finerenone treatment, reinforcing its potential for renal protection in CKD [29]. Our real-world study, focusing on finerenone's use in non-diabetic CKD patients, reveals encouraging results. A reduction in UACR was noted as early as 1 month into treatment, although this reduction was not statistically significant, likely due to the limited sample size. However, a more pronounced and statistically significant decrease was observed at the 3-month mark, signifying an effective amelioration of renal function. The previous study by Pitt et al. involved a cohort of heart failure patients with reduced ejection fraction (HFrEF) combined with mild-to-moderate CKD, of which two-thirds were non-diabetic [23]. In their study, a decrease in UACR was also observed 1 month after finerenone treatment across various dosage regimens. These findings showed a UACR reduction trend consistent with our study. However, the absence of a diabetes-specific subgroup analysis in Pitt's study limits the generalizability of their findings for non-diabetic CKD patients [23]. By addressing this gap, our study offers a more precise evaluation of finerenone in this population, thereby enriching the existing clinical evidence and enhancing our understanding of finerenone’s role in non-diabetic CKD management. While our research reveals encouraging results, there is a notable scarcity of direct evidence regarding the efficacy and safety of finerenone in non-diabetic CKD patients. This should be critically examined in large-scale randomized-controlled trials.

In CKD, renal function deterioration is typically characterized by a progressive decrease in eGFR [30]. Previous studies focusing on diabetic CKD reported that while finerenone could mitigate renal function decline, these patients still experienced an eGFR decrease. Several studies have also observed an initial downtrend in eGFR with commonly used CKD treatments like RASi [31], sodium-glucose cotransporter 2 inhibitors (SGLT-2i) [32], and non-steroidal MRAs [33]. This initial eGFR reduction is usually reversible and does not negate the long-term renal-protective effects of these therapies. For instance, the FIDELIO-DKD study reported a biphasic pattern in eGFR decline among diabetic CKD patients treated with finerenone [16]. Initially, a more pronounced eGFR reduction was observed in the finerenone group compared to placebo, but this trend reversed after 4 months, resulting in a slower eGFR decline in the finerenone group and a subsequent crossover in eGFR levels between the two groups [16]. In contrast, our study in non-diabetic CKD patients presents a different pattern of eGFR.

Notably, half of our study patients were concurrently on RASi drugs. Subgroup analysis, stratified by RASi use, revealed no substantial eGFR reduction over the 3-month follow-up period in either subgroup. This finding has significant implications, since a subset of patients in clinical practice face challenges with intolerance to RASi treatment due to blood pressure issues or adverse reactions. In the subgroup without concurrent RASi, finerenone treatment resulted in a notable reduction in UACR without eGFR deterioration over the 3-month follow-up. This suggests that direct initiation of finerenone treatment may be a feasible strategy for patients who cannot tolerate RASi therapy. In our study, no significant changes in eGFR were observed during the 3-month follow-up, indicating that no patients experienced acute kidney injury or renal failure after using finerenone. This stability in eGFR levels further supports the safety profile of finerenone in non-diabetic CKD patients. The observed stability in eGFR suggested that finerenone might be capable of protecting the renal function and retarding the progressive deterioration of eGFR. Finerenone might offer better glomerular protection in non-diabetic CKD compared to its effects in diabetic CKD, where varying degrees of eGFR decline have been reported. For example, the EVALUATE clinical trial [34] and a retrospective study [22] reported minimal changes in eGFR with another MRA, eplerenone, in non-diabetic CKD patients. This might also support that finerenone, or MRAs, might had a promising effect in maintaining eGFR in non-diabetic CKD. Limited by the risk of hyperkalemia related with other steroidal MRAs, the available evidence of MRAs in CKD is rather limited, and most are from studies with small sample size. The non-steroidal nature of finerenone endowed it with different pharmacological characteristics and warrant further exploration. However, the follow-up period in this study was relatively short (3 months), which may have limited the ability to fully evaluate long-term eGFR changes and other potential adverse effects. Therefore, the results for eGFR still need to be further validated in larger sample size studies.

These intriguing findings have sparked heightened interest in the ongoing FIND-CKD clinical trial (NCT05047263). The FIND-CKD trial is a randomized, double-blind, placebo-controlled, parallel-group, phase 3 study designed to investigate the efficacy and safety of finerenone in addition to standard care. It specifically focuses on the progression of kidney disease in patients with non-diabetic CKD. Participants in this trial will undergo a comprehensive evaluation over an extended period of 50 months, during which they will be randomly assigned to receive either oral finerenone or placebo. The primary endpoint of the FIND-CKD trial is defined as the mean rate of change, measured by the total slope of eGFR from baseline to 32 months. This extensive study duration and rigorous design can provide a more comprehensive understanding of finerenone's impact on renal function in non-diabetic CKD patients. Additionally, the crossover in eGFR levels observed in the FIDELIO-DKD study raises questions about the temporal dynamics of finerenone's impact on renal function. The FIND-CKD trial, with its prolonged follow-up and placebo-controlled design, is well positioned to contribute crucial information on the trajectory of eGFR changes and the overall renal-protective effects of finerenone in non-diabetic CKD. In brief, the preliminary findings from our study underscore the need for further investigation into finerenone's efficacy in non-diabetic CKD patients. The ongoing FIND-CKD trial holds the promise of elucidating the long-term impact of finerenone on renal function in this patient population.

The sK^+^ fluctuations observed in our study were rather subdued during the 3-month follow-up. This stability in sK^+^ levels, even with increased dosages in some patients, underscores finerenone’s safety in this context. Finerenone, as an MRA, influences sodium retention and potassium excretion, which can lead to hyperkalemia, especially in patients with CKD who already have compromised excretory function. Hyperkalemia is a specific concern for adverse event associated with patients receiving MRA drugs [23]. In clinical trials such as the ARTS-DN [15], FIDELIO-DKD [11], and FIGARO-DKD [18] studies, finerenone treatment was also associated with a modest increase in sK^+^ levels, particularly at higher doses, although severe hyperkalemia was rare. In the FIDELIO-DKD trial, hyperkalemia-related events doubled compared to placebo but did not result in fatal outcomes [11]. Similarly, the FIGARO-DKD study reported a higher incidence of hyperkalemia with finerenone, yet with minimal clinical complications [18]. These findings suggest that while finerenone treatment is linked to an increased risk of hyperkalemia, careful monitoring and dosage adjustment can effectively mitigate this risk. Additionally, no other adverse events related to finerenone were recorded during the treatment period. This absence of other adverse reactions, coupled with the stable sK^+^, reinforces the favorable safety profile of finerenone in this patient population.

This study has certain limitations. Retrospective analyses, while valuable for initial observations, are intrinsically limited by the absence of controlled variables and prospective planning, which can introduce potential bias in data interpretation. Moreover, the small sample size of our study further constrains the generalizability of our findings and limits the observation of rare adverse events with low incidence. Additionally, the relatively short duration of follow-up in our study presents another limitation. Short-term observations may not accurately capture long-term outcomes and potential adverse effects. The absence of a control group in our study design also limits the robustness of our findings. While the self-control comparison of clinical indicators effectively supports our research objective, including a control group would enhance the credibility of the results. To substantiate and expand upon our findings, future prospective studies or large-scale randomized-controlled trials with longer follow-up periods and control groups are warranted.

In summary, the findings in the present study suggest that finerenone leads to a significant reduction in UACR while maintaining stable eGFR in patients with non-diabetic CKD. The safety profile of finerenone was also favorable, with well-controlled sK^+^ levels. To the best of our knowledge, this study is the first to present real-world data on finerenone in non-diabetic CKD, offering novel insights and valuable clinical evidence for its use in this context. The forthcoming results from the FIND-CKD study are expected to further validate the efficacy and safety of finerenone in non-diabetic CKD, potentially shaping future treatment strategies in non-diabetic CKD management.

## Supplementary Information

Below is the link to the electronic supplementary material.

Supplementary file1 (DOCX 140 KB)

## Data Availability

The authors confirm that the data supporting the findings of this study are available within the article and its supplementary materials.
