# Supplementary material for: Effectiveness and safety of finerenone in Chinese CKD patients without diabetes: a retrospective, real-world study
Source: Int Urol Nephrol. 2024 Jul 10;56(12):3877–85. doi: 10.1007/s11255-024-04142-1 (PMC11534878; doi:10.1007/s11255-024-04142-1)
Supplement: Supplementary file 1 — Supplementary file1 (DOCX 140 KB) [file 11255_2024_4142_MOESM1_ESM.docx]

**Supplementary materials**

**Effectiveness and safety of finerenone in Chinese CKD patients without diabetes: a retrospective, real-world study**

**Table of content**

[Supplemental Table 1. Changes in urinary albumin-to-creatinine ratio (UACR) in patients with or without renin-angiotensin system inhibitor treatment. 2](#_Toc156910137)

[Supplemental Table 2. Changes in estimated glomerular filtration rate (eGFR) in patients with or without renin-angiotensin system inhibitor treatment. 3](#_Toc156910138)

[Supplemental Figure 1. Changes in urinary albumin-to-creatinine ratio (UACR) in patients with or without renin-angiotensin system inhibitor (RASi) treatment. 4](#_Toc156910139)

[Supplemental Figure 2. Changes in estimated glomerular filtration rate (eGFR) in patients with or without renin-angiotensin system inhibitor (RASi) treatment. 5](#_Toc156910140)

# Supplemental Table 1. Changes in urinary albumin-to-creatinine ratio (UACR) in patients with or without renin-angiotensin system inhibitor treatment.

| **RASi use** | **Baseline** | **Month 1** | **Difference from baseline** | **Difference from baseline (%)** | **Month 3** | **Difference from baseline** | **Difference from baseline (%)** |
| --- | --- | --- | --- | --- | --- | --- | --- |
| No |  |  |  |  |  |  |  |
| n (%) | 5 (62.5) | 3 (37.5) | 2 (25.0) | 2 (25.0) | 5 (62.5) | 2 (25.0) | 2 (25.0) |
| mean±SD | 2448.99±3275.59 | 1787.39±2605.20 | -38.88±33.98 | -10.03±12.35 | 981.59±1480.79 | -684.97±922.33 | -34.51±9.79 |
| median (IQR) | 295.92 (79.12, 4847.33) | 513.48 (288.88, 2648.95) | -38.88 (-50.89, -26.86) | -10.03 (-14.40, -5.67) | 237.80 (46.34, 1099.24) | -684.97 (-1011.06, -358.87) | -34.51 (-37.97, -31.05) |
| Yes |  |  |  |  |  |  |  |
| n (%) | 6 (75.0) | 4 (50.0) | 3 (37.5) | 3 (37.5) | 4 (50.0) | 4 (50.0) | 4 (50.0) |
| mean±SD | 1060.98±1074.13 | 384.62±561.49 | 113.02±407.21 | 4.17±73.59 | 196.04±199.41 | -461.75±626.58 | -60.77±29.04 |
| median (IQR) | 665.11 (502.68, 1267.22) | 147.74 (44.58, 487.78) | -21.37 (-115.69, 274.53) | -30.04 (-38.06, 29.30) | 152.03 (54.08, 293.99) | -200.41 (-511.58, -150.59) | -59.48 (-77.40, -42.85) |

SD, standard deviation; IQR, interquartile range.

# Supplemental Table 2. Changes in estimated glomerular filtration rate (eGFR) in patients with or without renin-angiotensin system inhibitor treatment.

| **RASi use** | **Baseline** | **Month 1** | **Difference from baseline** | **Difference from baseline (%)** | **Month 3** | **Difference from baseline** | **Difference from baseline (%)** |
| --- | --- | --- | --- | --- | --- | --- | --- |
| No |  |  |  |  |  |  |  |
| n (%) | 7 (87.5) | 6 (75.0) | 6 (75.0) | 6 (75.0) | 6 (75.0) | 5 (62.5) | 5 (62.5) |
| mean±SD | 80.52±36.11 | 79.59±36.75 | 1.98±4.80 | 4.86±6.96 | 77.37±29.91 | 0.89±13.70 | 10.03±29.84 |
| median (IQR) | 97.31 (59.06, 99.44) | 95.53 (52.33, 98.28) | 1.54 (-1.70, 3.47) | 5.40 (-1.58, 10.58) | 88.90 (64.16, 93.97) | 0.70 (-6.45, 3.74) | 2.65 (-6.63, 4.50) |
| Yes |  |  |  |  |  |  |  |
| n (%) | 7 (87.5) | 3 (37.5) | 3 (37.5) | 3 (37.5) | 3 (37.5) | 3 (37.5) | 3 (37.5) |
| mean±SD | 79.79±29.00 | 82.97±43.76 | -1.82±5.32 | -3.63±6.38 | 95.60±17.96 | -2.57±1.83 | -2.52±1.59 |
| median (IQR) | 81.60 (61.87, 102.62) | 100.02 (66.63, 107.83) | -3.08 (-4.73, 0.47) | -6.01 (-7.24, -1.20) | 101.79 (88.58, 105.72) | -1.97 (-3.29, -1.54) | -1.76 (-3.05, -1.61) |

SD, standard deviation; IQR, interquartile range.


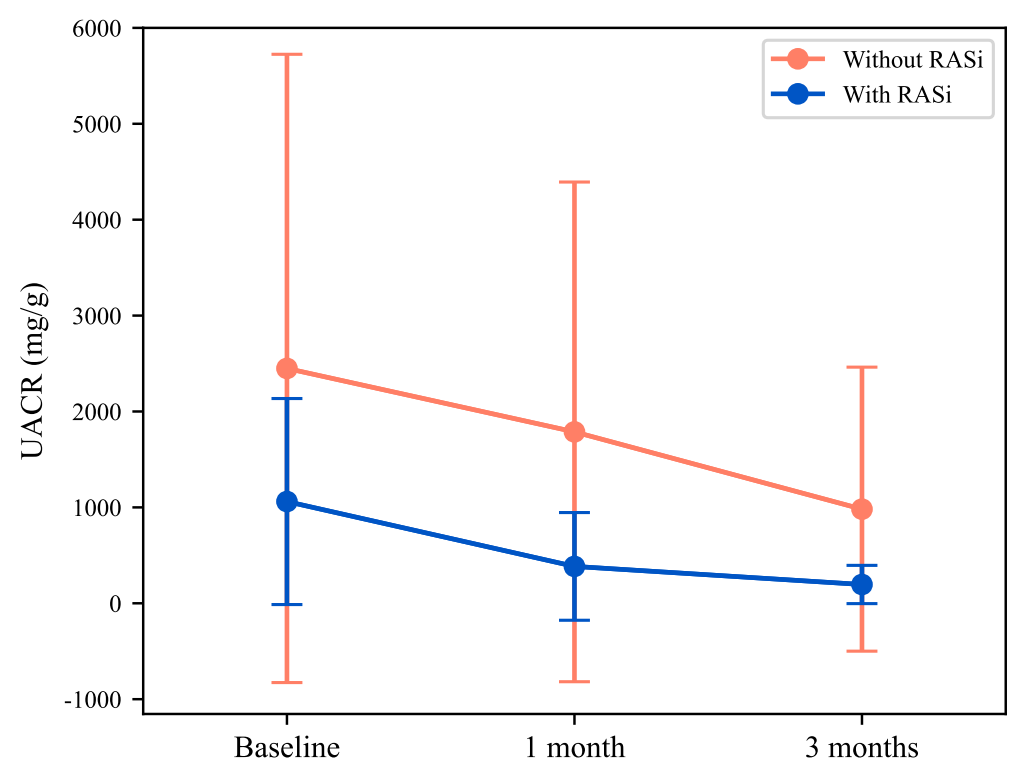


# Supplemental Figure 1. Changes in urinary albumin-to-creatinine ratio (UACR) in patients with or without renin-angiotensin system inhibitor (RASi) treatment.


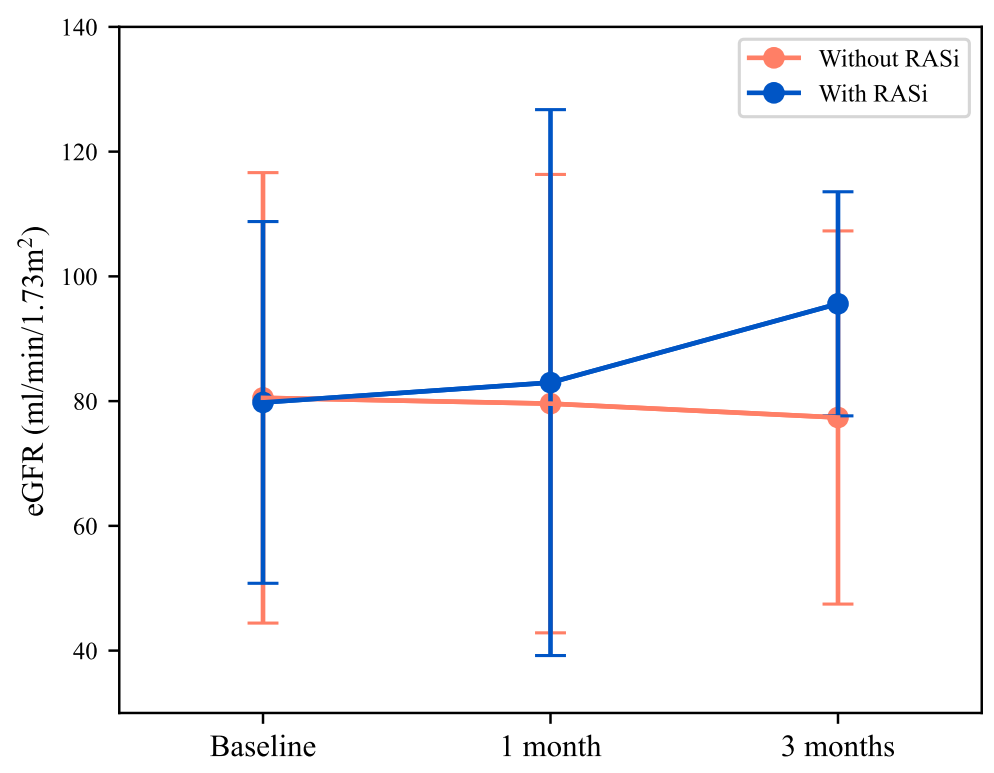


# Supplemental Figure 2. Changes in estimated glomerular filtration rate (eGFR) in patients with or without renin-angiotensin system inhibitor (RASi) treatment.
